# Seeds of Destruction: Predicting How microRNAs Choose Their Target

**DOI:** 10.1371/journal.pbio.0030114

**Published:** 2005-02-15

Compare the gene number of fruitfly (13,000) to human (20,000), and it's pretty clear that complexity emerges not just from gene number but from how those genes are regulated. In recent years, it's become increasingly clear that one class of molecules, called microRNAs (miRNAs), exert significant regulatory control over gene expression in most plant and animal species. A mere 22 nucleotides long, miRNAs control a cell's protein composition by preventing the translation of protein-coding messenger RNAs (mRNAs). When a miRNA pairs with an mRNA, through complementary base pairing between the molecules, the mRNA is either destroyed or is not translated.

Hundreds of miRNAs have been found in animals, but functions for just a few have been identified, mostly through genetic studies. Many more functions could be assigned if miRNA targets could be predicted. This approach has worked in plants, because miRNAs and their targets pair through the near perfect complementarity of their base pairs. But the molecules follow different rules in animals—duplexes contain just short stretches of complementary sequence interrupted by gaps and mismatches—which makes predicting miRNA targets a challenge.

In a new study, Stephen Cohen and his colleagues at the European Molecular Biological Laboratory in Germany establish basic ground rules for miRNA–mRNA pairing using a combination of genetics and computational analyses, and identify different classes of miRNA targets with distinct functional properties. Although the miRNA is only 22 nucleotides long, its 5′ and 3′ ends seem to have distinct roles in binding. Cohen and colleagues show that miRNA functional targets can be divided into two broad categories: those that depend primarily on pairing to the miRNA's 5′ end (called 5′ dominant sites), with varying degrees of 3′ pairing, and those that also need the miRNA's 3′ end (called 3′ compensatory sites). Surprisingly, miRNAs can regulate their targets simply by strong pairing with so-called seed sites that consist of just seven or eight bases complementary to the miRNA 5′ end. Target sites with weaker 5′ complementarity need supplemental pairing with the miRNA's 3′ end to function. The finding that so little sequence complementarity is needed means that there are many more target sites than had been previously recognized.

The miRNA 3′ end, while not essential, is expected to confer some function, since it tends to be conserved in animals—miRNA 3′ ends provide an additional measure of regulatory control by permitting the function of target sites that have only limited complementarity to the miRNA 5′ end. The authors speculate that seed sites might be the first functional sites acquired by protein-coding genes that require repression, and that additional sites might be acquired to promote stronger repression.

Based on their experimental results, Cohen and colleagues searched the Drosophila genome for biologically relevant targets, and estimate that the fly has about 100 sites for every miRNA in its genome. Since the fruitfly has anywhere from 96 to 124 miRNAs, that means it has 8,000 to 12,000 target sites (in the 11,000 genes sampled). This indicates that miRNAs regulate a large fraction of protein-coding genes. Of the known animal miRNAs, many regulate critical developmental processes. This new approach to predicting targets should help reveal just how much regulatory control actually flows from these tiny bits of RNA.

